# Predicting Children's Reading and Mathematics Achievement from Early Quantitative Knowledge and Domain-General Cognitive Abilities

**DOI:** 10.3389/fpsyg.2016.00775

**Published:** 2016-05-25

**Authors:** Felicia W. Chu, Kristy vanMarle, David C. Geary

**Affiliations:** ^1^Department of Psychological Sciences, University of MissouriColumbia, MO, USA; ^2^Interdisciplinary Neuroscience Program, University of MissouriColumbia, MO, USA

**Keywords:** mathematics achievement, reading achievement, quantitative abilities, preschool, domain-specific abilities, domain-general abilities

## Abstract

One hundred children (44 boys) participated in a 3-year longitudinal study of the development of basic quantitative competencies and the relation between these competencies and later mathematics and reading achievement. The children's preliteracy knowledge, intelligence, executive functions, and parental educational background were also assessed. The quantitative tasks assessed a broad range of symbolic and nonsymbolic knowledge and were administered four times across 2 years of preschool. Mathematics achievement was assessed at the end of each of 2 years of preschool, and mathematics and word reading achievement were assessed at the end of kindergarten. Our goals were to determine how domain-general abilities contribute to growth in children's quantitative knowledge and to determine how domain-general and domain-specific abilities contribute to children's preschool mathematics achievement and kindergarten mathematics and reading achievement. We first identified four core quantitative competencies (e.g., knowledge of the cardinal value of number words) that predict later mathematics achievement. The domain-general abilities were then used to predict growth in these competencies across 2 years of preschool, and the combination of domain-general abilities, preliteracy skills, and core quantitative competencies were used to predict mathematics achievement across preschool and mathematics and word reading achievement at the end of kindergarten. Both intelligence and executive functions predicted growth in the four quantitative competencies, especially across the first year of preschool. A combination of domain-general and domain-specific competencies predicted preschoolers' mathematics achievement, with a trend for domain-specific skills to be more strongly related to achievement at the beginning of preschool than at the end of preschool. Preschool preliteracy skills, sensitivity to the relative quantities of collections of objects, and cardinal knowledge predicted reading and mathematics achievement at the end of kindergarten. Preliteracy skills were more strongly related to word reading, whereas sensitivity to relative quantity was more strongly related to mathematics achievement. The overall results indicate that a combination of domain-general and domain-specific abilities contribute to development of children's early mathematics and reading achievement.

## Introduction

Numeracy and literacy at school completion predict employability and wages in adulthood (Bynner, 2004), and basic quantitative and preliteracy skills at school entry presage numeracy and literacy at school completion (Duncan et al., 2007). Identifying the factors that contribute to poor school entry mathematics and reading achievement has the potential to inform early remediation approaches for at-risk children. Research to date indicates that preschoolers' phonological awareness and letter knowledge predict later reading abilities (e.g., Lonigan et al., 2000; for a review and meta-analysis, see Wagner and Torgesen, 1987; Melby-Lervåg et al., 2012), but the research base on early quantitative skills and later mathematics achievement is not nearly as extensive (cf. Hannula and Lehtinen, 2005; Jordan et al., 2007). In addition to research on domain-specific predictors of later literacy and numeracy, there is a growing literature on the similarities and differences in the factors that predict growth in mathematics and reading achievement, but these studies have focused on elementary-school children (e.g., Koponen et al., 2007, 2013; Geary, 2011; Fuchs et al., 2016). Our study extends previous research by including a more extensive assessment of symbolic and nonsymbolic quantitative knowledge than in most previous studies, and by focusing on children who are younger than those in most previous studies. With this approach, we identify the beginning of preschool competencies that predict mathematics achievement and achievement growth over 2 years of preschool, and the similarities and differences in the competencies that predict mathematics and reading achievement at the end of kindergarten.

### Similarities and differences in predictors of mathematics and reading achievement

It is now recognized that growth in mathematics and reading competencies are related. In a large-scale longitudinal study, Grimm (2008) showed that third graders' reading comprehension predicted growth in several mathematical areas through eighth grade. It is not clear however whether the relation was due to reading competence *per se* or whether performance on the reading comprehension measure was a proxy for individual differences in domain-general abilities, such as working memory and intelligence, that predict achievement growth across academic domains. In any case, other studies have also found similarities as well as differences in the brain and cognitive systems that support children's reading and mathematical development (Mann Koepke and Miller, 2013; Willcutt et al., 2013; for a review, see Ashkenazi et al., 2013).

As an example of basic brain and cognitive processes that might be common across academic domains, consider that children's ability to rapidly name stimuli (e.g., letters, numbers, colors), measured by Rapid Automatized Naming (RAN) tasks, has been found to predict mathematics and reading achievement. In a kindergarten to eighth grade longitudinal study, Mazzocco and Grimm (2013) found that children with mathematical learning disabilities and reading disabilities both suffered from deficits in RAN performance. Similarly, mathematics and reading skills can be predicted by earlier RAN performance (Koponen et al., 2007, 2013) and some aspects of counting skill, such as counting by 2 s (Koponen et al., 2013). Koponen et al. (2013) suggested their tasks predicted both reading and mathematics performance because they reflected the ease of forming and retrieving arbitrary visual-verbal associations in long-term memory. Individual differences in ease of forming and retrieving these associations may depend on the functional integrity of the hippocampal-dependent memory system that is a domain-general system for associative learning and potentially a linchpin for aspects of children's cognitive development across academic domains (Qin et al., 2014).

More complex domain-general abilities that are often found to predict achievement across domains include, as noted, intelligence (or reasoning abilities), the central executive component of working memory (or executive functions), and in-class attentiveness (e.g., Hoge and Luce, 1979; Clark et al., 2010; Geary, 2011; Fuchs et al., 2016). For example, preschoolers with stronger executive functions showed greater gains in mathematics and reading achievement over the first 3 years of elementary school than their peers with weaker early skills (Bull et al., 2008). Working-memory deficits have also been found to be associated with both reading and mathematics learning disabilities (Geary, 1993, 2004; De Weerdt et al., 2013; Willcutt et al., 2013). The relative importance of these domain-general competencies may vary with the novelty and complexity of the achievement domain and with individual children's level of domain-specific expertise. For instance, in analyses of only domain-general competencies, Geary (2011) found that intelligence predicted school-entry mathematics and reading achievement and grade-to-grade gains in mathematics but not reading achievement. Fuchs et al. found that first graders' intelligence (reasoning), central executive, and in-class attentive behavior predicted arithmetic but not reading achievement in third grade, controlling domain-specific competencies. One possibility is that these domain-general competencies are particularly important for comprehending and learning novel material and become less important as individuals gain domain-specific expertise (Geary, 2005; Tricot and Sweller, 2014; Sweller, 2015). In this view, domain-general competencies may decline in importance as children become more efficient word readers, for instance, but these competencies remain predictive of mathematics because the curriculum and associated achievement tests are continually adding more complex material; word reading tests become more difficult but largely because the more difficult words are low frequency and not because conceptually novel material is added to the associated achievement tests.

As an example of domain-specific expertise, consider Geary's (2011) first-to-fifth grade longitudinal study of quantitative and domain-general cognitive predictors of mathematics achievement and word reading achievement. The only quantitative predictor of school-entry word reading skill was simple addition retrieval, which is dependent on associative learning (Siegler and Shrager, 1984; Qin et al., 2014). The remaining mathematical cognition predictors were unique to mathematics. For instance, early fluency in combining the cardinal value of collections of objects with the cardinal value of Arabic numerals predicted school-entry mathematics but not reading achievement, and children's early knowledge of the mathematical number line predicted growth in mathematics achievement through fifth grade but was unrelated to reading achievement in any grade. In a similar study, Fuchs et al. (2016) found that first graders' phonological memory (e.g., memory for word sounds) and general language competencies predicted reading but not arithmetic achievement 2 years later, controlling working memory, intelligence, and in-class attentive behavior. Children's early skill at identifying letters and simple high-frequency words predicted later word reading and arithmetic achievement, but the magnitude of the effect was 3.5 times stronger for reading than arithmetic. Knowledge of addition facts in second grade predicted word reading and arithmetic achievement in third grade but the effect was 3.5 times larger for arithmetic than reading. Addition retrieval is based on the hippocampal-dependent memory system (Qin et al., 2014) and thus will index ease of forming the visual-verbal associations that are important across reading and mathematics, but is also domain-specific content knowledge for arithmetic but not reading. Similarly, letter and word identification will index individual differences in the same memory system, but is also domain-specific content knowledge for reading but not arithmetic.

In other words, domain-general cognitive and learning systems will influence the acquisition of domain-specific knowledge and thus may be correlated with achievement in unrelated domains. For the latter, the correlations will then reflect the prior influence of domain-general systems and not the importance of content-specific knowledge *per se*. Isolating knowledge and competencies that are specific to one domain (e.g., mathematics) or another (e.g., reading) will thus require simultaneous estimation of the effects of domain-general abilities on each domain and demonstration that content-specific knowledge influences achievement in one domain but not the other.

### Longitudinal predictors of mathematics and reading achievement

#### Mathematics achievement

As previously mentioned, our study includes an extensive, longitudinal assessment of symbolic and nonsymbolic quantitative competencies. Early symbolic knowledge involves learning counting words and Arabic numerals and the quantities they represent (i.e., their cardinal values) and the relations between them (e.g., 4 > 3; Fuson, 1988; Wynn, 1990; Geary, 1994; Le Corre and Carey, 2007). Early nonsymbolic competencies are largely supported by the evolutionarily ancient approximate number system (ANS) that supports an intuitive understanding of the relative magnitudes of collections of items and the ability to manipulate (e.g., add) the associated representations (for reviews, see Feigenson et al., 2004; Geary et al., 2015).

At this time, there is lively debate over the relative importance of early symbolic vs. nonsymbolic magnitude processing for later mathematics achievement. There is evidence that acuity of the ANS, allowing for fine discriminations among nonsymbolic quantities, is correlated with retrodictive, concurrent, and prospective mathematics achievement, controlling working memory and intelligence (e.g., Halberda et al., 2008; Libertus et al., 2011; Mazzocco et al., 2011). However, in a qualitative review of existing studies, De Smedt et al. (2013) concluded there was more consistent evidence for the role of symbolic than nonsymbolic knowledge in predicting mathematics achievement. Schneider et al.'s (2016) recent meta-analysis supports this conclusion, but also provided evidence for a small (*r* ~ 0.2) but consistent relation between measures of ANS acuity and mathematics achievement, in keeping with several earlier meta-analyses (Chen and Li, 2014; Fazio et al., 2014). Several studies suggest more nuanced relations among nonsymbolic and symbolic quantitative competencies and mathematics achievement; specifically, that ANS acuity contributes to the ease of learning the cardinal value of number symbols, which then becomes critical for children's mathematical development (vanMarle et al., 2014; Chu et al., 2015). In any case, the unsettled state of the field necessitates the inclusion of both symbolic and nonsymbolic quantitative tasks in the study of the foundations of children's mathematics achievement and growth in this achievement.

As noted, there is also evidence that preschoolers' executive functions contribute to growth in mathematical competencies (e.g., Blair and Razza, 2007; Bull et al., 2008; Clark et al., 2010). Executive functions and the central executive component of working memory are composed of subskills that may be differentially related to mathematical competencies at different ages. These include the ability to suppress prepotent responses (inhibition), shift attention between tasks (shifting), and explicitly monitor and update information (updating) represented in the phonological loop or visuospatial sketchpad (Miyake et al., 2000). Although updating may be critical for older children (Bull and Lee, 2014), inhibitory control may be especially important for young children's mathematical development (Espy et al., 2004; Blair and Razza, 2007; Fuhs and McNeil, 2013). Several studies have found a relation between inhibitory control and preschoolers' and kindergartners' mathematics achievement, even with control of other factors (e.g., child age, verbal intelligence, maternal education, and other components of executive functions; Espy et al., 2004; Blair and Razza, 2007; Clark et al., 2010).

Fuhs and McNeil (2013) argued that inhibitory control may also be related to children's nonsymbolic magnitude processing. In a study of preschoolers from low-income homes, they found that inhibitory control influenced the strength of the association between nonsymbolic magnitude processing and mathematics ability; specifically, the relation between one component of nonsymbolic quantity processing and mathematics achievement was no longer significant after controlling children's inhibitory control. Keller and Libertus (2015) however found that the relation between measures of ANS acuity and mathematics achievement were significant above and beyond the influence of inhibitory control. The issue of whether or not the relation between ANS acuity and mathematics achievement is mediated by inhibitory control remains to be determined, but the importance of executive functions generally, and inhibitory control in particular, for younger children's mathematics development is clear.

Previous research has also shown that on average, children from lower SES families have lower number knowledge and mathematics achievement than children from middle to high SES families (e.g., Klibanoff et al., 2006). One potential mediating factor is parent education that in turn is related to use of mathematical language in the home (e.g., Levine et al., 2010; McNeil et al., 2011; Purpura and Reid, 2016). Levine et al. found that children whose parents engaged in more number talk (e.g., counting, referring to cardinal values) learned the cardinal values of number words earlier than children of less verbose parents, independent of SES (measured by parent education and income). Generally, however, parents from higher SES homes are more likely to engage in set size comparisons and calculations with their children than parents from low SES families, whereas parents from low SES families engage in more rote counting, numeral recognition, and labeling of numerosities. In other words, even when less educated parents engage in number talk with their children, it is less sophisticated than that of higher-SES parents. Better educated parents also have higher expectations for their children and a better understanding of skills within the child's developmental range (Purpura and Reid, 2016). The latter is consistent with Davis-Kean's (2005) finding that parents who were more educated were better able to adjust their expectations to match their child's ability level. Whatever the mechanisms, these studies indicate that parental education should be controlled when attempting to isolate the contributions of early nonsymbolic and symbolic quantitative competencies on later mathematics achievement.

#### Reading achievement

There is evidence that young children's letter knowledge and phonological awareness predict their later reading achievement (e.g., Bond and Dykstra, 1967; Wagner and Torgesen, 1987; Badian, 1994; Lonigan et al., 2000; Melby-Lervåg et al., 2012). Bond and Dykstra, for example, found that young children's ability to recognize letters of the alphabet was the single best predictor of their reading achievement in first grade; phonemic awareness was the second best early predictor. Melby-Lervåg et al. suggested that letter naming ability contributes significantly to reading development and is partly independent of the effects of phonological awareness and phonological processing. The contribution of letter knowledge is related to children's ability to understand the mapping between the letters in written words and the phonemes in spoken language, but they must first know the names of letters.

We thus decided to include a measure of letter knowledge in our study as a potential contrast to quantitative knowledge in predicting later achievement. It would have been preferable to include a wider range of preliteracy measures, especially of phonemic awareness, but based on our focus on mathematical development and constraints on how often we could assess children, we could include only a single measure.

### Present study

Previous studies have largely focused on elementary school children, and to date, have not thoroughly explored differences in how preschool children's quantitative and preliteracy competencies predict later mathematics and reading achievement. In addition to a broad assessment of nonsymbolic and symbolic quantitative competencies and letter knowledge, we assessed the key domain-general abilities of intelligence and executive functions, as well as parental educational background. After identifying the core quantitative abilities that predict later achievement, we document the relation between domain-general abilities, parental background, and preliteracy skills and gains in these quantitative competencies across 2 years of preschool. We then focus on the relation between beginning of preschool quantitative and preliteracy skills and mathematics achievement across the preschool years and mathematics and reading achievement at the end of kindergarten. In all, the study allowed us to identify core predictors of early gains in mathematics achievement and similarities and differences in the predictors of later mathematics and reading achievement.

## Materials and methods

### Participants

One hundred and fifty-three children (71 boys) were recruited from the Title I preschool program within the public school system in Columbia, Missouri, in the Midwestern United States. Title I preschool is a federally funded program that offers services to 3- to 5- year olds who may be at risk for academic failure. Children were recruited in two cohorts, entering in the fall of 2011 and 2012, and completed assessments during 2 years of preschool and 1 year of Kindergarten. Of the original sample, 107 children had IQ scores >70 (6 had scores < 70) and completed all of the testing sessions during both years of preschool and kindergarten. Of these 107 children, 7 of them failed to complete multiple tasks within one or more testing sessions (due largely to inattention) and thus they were also dropped from analyses. The final sample included 100 (44 boys) children whose age at the time of the first assessment was 3 years 10 months (ranging from 3 years 2 months to 4 years 4 months) and 6 years 2 months (ranging from 5 years 7 months to 6 years 10 months) at the time of the final assessment.

Demographic information was obtained through parent survey for a subset of the sample (*n* = 88). This survey included questions on children's and parents' racial and ethnic background, as well as parents' education level, household income, housing (rent/own, monthly rent/mortgage), and whether they received food or housing assistance. The ethnic composition was 83% non-Hispanic, 13% Hispanic/Latino; 4% of parents did not respond to the ethnicity question. The racial composition was 61% White, 20% Black, 13% mixed race, and 6% Asian. The self-reported total household income was: $0 to $25,000 (33%), $25,000 to $50,000 (24%), $50,000 to $75,000 (26%), $75,000 to $100,000 (14%), $100,000 to $150,000 (1%), and $150,000 or above (1%). Seven percent of respondents reported receiving housing assistance, and 37% reported receiving food stamps.

As a proxy for socioeconomic status, we used parental education level. Mother's education and father's education were highly correlated (*r* = 0.63, *p* < 0.001), and thus these variables were collapsed into a parental education category consisting of three levels (no information, high school diploma or less, or at least a college degree) based on the highest attainment of the two parents. Forty-three percent of the children had at least one parent with a high school diploma, and 40% had at least one parent with a college degree. Parental education level was unknown for the remaining 17% of the children.

### Materials

#### Quantitative tasks

As described below, the children were administered 12 quantitative tasks multiple times during preschool, but with data reduction analyses we reduced this to four key tasks: *give-a-number, discrete quantity discrimination, numeral recognition*, and *nonverbal calculation*. These four quantitative tasks are described below in more detail, while the remaining tasks are described briefly.

##### Give-a-number

The give-a-number task is frequently used to measure how well children understand the cardinal values of number words (Wynn, 1990). In the task, children are asked to feed a puppet by placing the requested number of “cookies” (1, 2, 3, 4, 5, or 6) on a plate. All children started at set size 1. Set size increased by 1 following correct answers and decreased by 1 following incorrect answers. The highest number of objects accurately given to the experimenter on at least two out of three attempts was taken as the highest set size for which the child understood cardinality (Le Corre and Carey, 2007).

##### Numeral recognition

The children were shown cards with an Arabic numeral (ranging from 1 to 15) and asked to name the numeral. The cards were presented in a random order and the score was the number of numerals correctly named.

##### Discrete quantity discrimination

The children completed a commonly used assessment of ANS acuity, the discrete quantity discrimination task, using the Panamath program (Halberda et al., 2008). For this task, two separate arrays of blue and yellow dots were presented simultaneously on a screen for 2533 ms (to discourage counting), and the children were asked to identify which set “had more dots” (by pointing or by saying “blue” or “yellow”). All dot displays consisted of 5–21 dots. Dot size varied to keep the total area of the dots constant across the two arrays for half of the trials. Children in the first cohort received 24 test trials, and based on low performance for some children in this cohort, 6 relatively easy trials were added for the second cohort and for both cohorts in the second year. On each trial, the ratio of blue:yellow dots was determined randomly on each trial. For the original 24 trials, this ranged between 1.29 and 3.38, and the 6 added trials ranged between 3.5 and 4.0. Scores on this task were determined by accuracy (percent correct out of all trials), which has been shown to be a more reliable measure of ANS acuity for young children than the Weber fraction, which describes the degree of variability in the underlying representations (Inglis and Gilmore, 2014). The Spearman-Brown prediction formula applied to the correlation between percentage correct for the first and second halves of the items provided a reliability estimate of 0.87 for the first cohort and 0.71 for the second cohort in their first year of preschool. For the second year of preschool, the reliability estimates were 0.90 and 0.81 for the first and second cohorts, respectively.

##### Nonverbal calculation

To measure children's non-symbolic arithmetic abilities, children were shown the addition or subtraction of one or more disks from a hidden set of disks and asked to predict the exact numerical result (Levine et al., 1992; Huttenlocher et al., 1994). Children watched an experimenter place some disks on a mat. The experimenter then covered the disks with a plate, and then added or subtracted disks from under the plate. After the transformation, children were asked to generate the (hidden) result on their mat. After four familiarization trials in which children simply matched a hidden set, children completed 12 test trials, presented in random order: 3−1, 2+2, 4−2, 1+3, 4−1, 4+1, 3+2, 1+4, 5−2, 5−3, 2+4, and 6−4. The score was the percentage of correct trials out of trials attempted.

##### Remaining quantitative tasks

The remaining quantitative tasks included enumeration, point-to-x, magic box, ordinal choice, verbal counting, numeral comparison, counting knowledge, and continuous quantity discrimination. The enumeration task involved counting an array of 20 stickers. For the point-to-x task, children saw two sets of pictured objects displayed on a laptop and identified which side of the screen contained x objects. The magic box task was a variant of Starkey's (1992) search-box task and assessed children's implicit understanding of addition and subtraction for set sizes less than four. For the ordinal choice task, children watched as the experimenter dropped objects in one cup and then dropped objects in another cup. The children were then asked to identify, without counting, which cup had more objects. Children counted as high as they could (starting from 1) in the verbal counting task. In the numeral comparison task, children were shown pairs of cards (from correctly identified numerals in the numeral recognition task) and asked to identify the larger numeral. The counting knowledge task assessed children's understanding of the counting principles (Gelman and Gallistel, 1978); they watched a puppet count in ways that were consistent with or violated counting principles and then indicated if the puppet counted correctly or incorrectly. The continuous quantity discrimination task was similar to the discrete quantity discrimination task, but children viewed a large rectangle that was composed of red and blue portions and were asked to identify whether there was “more red” or “more blue” in the picture.

#### Cognitive and achievement measures

##### Intelligence

The children completed three subtests of the Wechsler Preschool and Primary Scale of Intelligence-III (WPPSI; Wechsler, 2002); Receptive Vocabulary, Block Design, and Information. Following standard procedures, scores were scaled and prorated to generate an estimate of full scale IQ. The mean performance of the children here was average (*M* = 98, *SD* = 16).

##### Executive functions

Executive functions was assessed using the Conflict Executive Function (EF) scale developed for 2- to 6-year-olds (Beck et al., 2011; Carlson, 2012). The Conflict EF scale consists of a card-sorting task. Children were presented with two boxes with target cards affixed to the front. They were given a rule and asked to place a card in the appropriate box. There were 7 levels with 10 trials each (the first four included two sublevels of five trials each). Each level consisted of normal sorting trials, followed by conflict trials in which children sorted cards according to the opposite rule. For example, children first placed “big kitties” in the “big kitty” box and “little kitties” in the “little kitty” box; on conflict trials, they were asked to place, for example, “big kitties” in the “little kitty” box. For later trials, children sorted the cards depending on shape or color of the card (the rule was reversed for conflict trials). More advanced trials required children to sort cards according to their shape or color, depending on whether a black border was present or absent on the card. In order to advance to the next sublevel, children had to complete four out of five trials correctly, and in levels with 10 trials, children had to complete four shape trials and four color trials correctly in order to advance. The score was the total number of correct trials (max score = 70).

##### Preliteracy skills

Early preliteracy skills were assessed using the Upper-Case Alphabet Recognition subtest of the Phonological Awareness Literacy Screening-PreK (PALS; Invernizzi et al., 2004). For this task, children identified the upper-case alphabet letters on a card, and the score was the total number of letters correctly identified.

##### Preschool mathematics achievement

To measure mathematics achievement, children completed the Test of Early Mathematics Ability-3 (TEMA-3; Ginsburg and Baroody, 2003). This standardized test consists of items that require producing finger displays to represent different quantities, counting, making numerical comparisons, and using some informal arithmetic. Children started on the first item of the test and continued until they failed five consecutive items. Achievement was in the average range for the first (*M* = 93, *SD* = 14) and second (*M* = 95, *SD* = 14) year of preschool. To make scores comparable across years, the raw score was divided by the maximum score for our sample for each year. We chose to use the maximum score for our sample rather than the maximum possible score because the TEMA-3 is designed for children ages 3–8, and our children were on the younger side of the target age range. Thus, they were unlikely to achieve the maximum possible score.

##### Kindergarten achievement tests

During the spring semester of kindergarten, children completed the Numerical Operations and Word Reading subtests of the Wechsler Individual Achievement Test (WIAT; Wechsler, 2001). The achievement of the sample was in the average range for Numerical Operations (*M* = 107, *SD* = 12) and Word Reading (*M* = 112, *SD* = 13). To make the scores comparable across tests, we used the raw score divided by the maximum score for our sample on each subtest. We again chose to use the maximum score of our sample rather than maximum possible score because our children were on the younger side of the target age range of the tests.

The Numerical Operations test included items that assessed children's knowledge of counting and arithmetic. Simpler problems involved single digit addition and subtraction, while more complex problems involved multi-digit addition and subtraction. The Word Reading test assessed children's knowledge of letters, letter sounds, and ability to read common words. Words became more difficult further into the test. Following standard procedure, the Numerical Operations test was discontinued when children answered six consecutive questions incorrectly, and the Word Reading test was discontinued after children failed seven consecutive items.

### Procedure

As shown in Table 1, the children participated in six assessments during each of the 2 years of preschool and two assessments in kindergarten. For all assessments, children were tested individually in a quiet location at their preschool or kindergarten. The quantitative tasks were administered in two sessions in the fall and spring semesters of the preschool year and a different set of quantitative measures (not reported here) during the fall of kindergarten. The domain-general and preliteracy measures were administered at the beginning of the spring semester; the IQ test was only administered in the first year of preschool. At the end of each year of preschool, children completed the TEMA-3 and the WIAT at the end of kindergarten. Each test session lasted 20–40 min. The experimental procedure was reviewed and approved by the Institutional Review Board of the University of Missouri. Written consent was obtained from all parents, and all participants provided verbal assent for all assessments.

**Table 1:** **Sequence of tasks and ages**.

**Sequence of tasks**	**Age of children**
**YEAR 1 PRESCHOOL**	
Quant 1 (Fall) – Enumeration – Give-a-Number – Point-to-X – Magic Box – Discrete Quantity Discrimination – Ordinal Choice	Mean: 3 years 10 months Range: 3 years 2 months −4 years 4 months
Quant 2 (Fall) – Verbal Counting – Nonverbal Calculation – Numeral Recognition – Numeral Comparison – Counting Knowledge – Continuous Quantity Discrimination	Mean: 3 years 11 months Range: 3 years 4 months–4 years 4 months
Cognitive battery (Spring) – Executive Functions (Card Sorting) – WPPSI-III (Receptive Vocabulary, Block Design, Information) – Preliteracy (Upper-Case Alphabet Recognition)	Mean: 4 years 1 month Range: 3 years 5 months–4 years 8 months
Mathematics Achievement (Spring; Test of Early Mathematics Ability-3; TEMA-3)	Mean: 4 years 4 months Range: 3 years 9 months–4 years 10 months
**YEAR 2 PRESCHOOL**	
Cognitive battery (Spring) – Executive Functions	Mean: 5 years 0 months Range: 4 years 5 months–5 years 6 months
Quant 1 (Spring)	Mean: 5 years 2 months Range: 4 years 7 months–5 years 8 months
Quant 2 (Spring)	Mean: 5 years 3 months Range: 4 years 8 months–5 years 10 months
Mathematics Achievement (Spring; TEMA-3)	Mean: 5 years 4 months Range: 4 years 9 months–5 years 10 months
**KINDERGARTEN**	
WIAT (Spring)—Numerical Operations; Word Reading	Mean: 6 years 2 months Range: 5 years 7 months–6 years 8 months

*TEMA-3, Test of Early Mathematics Ability-3 (Ginsburg and Baroody, 2003); WIAT, Wechsler Individual Achievement Test (Wechsler, 2001)*.

### Analyses

Although the children completed the vast majority of tasks, 2% of the data were missing. These missing values were imputed using the multiple imputations procedure in SAS 9.4 (SAS Institute, 2012).

#### Variable reduction

To reduce the number of variables in the main analyses, and thus reduce false positives, we used a Bayes factor analysis using the “BayesFactor” program (Morey and Rouder, 2015) in R to determine the best quantitative predictors of each kindergarten achievement test. These analyses identified give-a-number, discrete quantity discrimination, nonverbal calculation, and numeral recognition as the best predictors of Numerical Operations, and give-a-number and numeral recognition as the best predictors of Word Reading. Thus, we only included the give-a-number, discrete quantity discrimination, nonverbal calculation, and numeral recognition tasks in our main analyses.

#### Main analyses

In the first of three sets of analyses, we examined beginning (i.e., fall semester of Year 1) to end (i.e., spring semester of Year 2) of preschool change in the four quantitative tasks identified above. Second, we examined the relation between beginning of preschool performance on these four variables and mathematics achievement across the 2 years of preschool, controlling for parental education, and beginning of preschool IQ, EF, and preliteracy skills. Finally, we redid these analyses substituting WIAT scores for TEMA-3 scores, focusing on the differential relations between early quantitative knowledge and mathematics and reading achievement at the end of kindergarten. All analyses were conducted using the PROC MIXED procedure in SAS 9.4 (SAS Institute, 2012). Intercept values were random effects, and predictor variables were standardized (*M* = 0, *SD* = 1).

Following Geary (2011), the first set of analyses included domain-general predictors (i.e., parental education, Y1 and Y2 EF, IQ, and preliteracy) and interactions with year. Interactions with *p* > 0.10 were dropped, starting with the highest *p*-value, and the model was re-estimated. The procedure continued until all interactions were significant. The iterative procedure was then continued for main effects. In the analyses predicting preschool mathematics achievement, the first step involved specifying a model using only the domain-general predictors and their interaction with preschool year. The same iterative procedure was followed until only significant interactions were remaining in the model; all main effects for the domain-general predictors were retained because they are standard covariates. Next, the four quantitative predictors and their interactions with preschool year were added to the final domain-general model. The same iterative procedure was then implemented for the quantitative variables. The same approach was used in the analyses of kindergarten achievement.

## Results

Descriptive information (min, max, mean, and SD) for the different tasks and measures are provided in Table 2, and correlations are given in Table 3.

**Table 2:** **Descriptive statistics for tasks assessed**.

**Variable**	**Mean**	**SD**	**Min**	**Max**
**YEAR 1**				
Give-a-number	3.38	1.88	0	6
Numeral recognition	4.76	4.28	0	15
Nonverbal calculation	23.30	16.83	0	66.67
Discrete quantity discrimination	66.80	16.53	36.67	100
EF	32.67	13.87	11	69
IQ	98.06	15.60	73	135
Preliteracy (alphabet knowledge)	12.97	9.18	0	26
Mathematics achievement	92.69	14.44	68	129
**YEAR 2**				
Give-a-number	5.57	1.05	2	6
Numeral recognition	9.76	3.98	0	15
Nonverbal calculation	44.96	21.46	0	91.67
Discrete quantity discrimination	86.04	16.36	43.33	100
EF	46.02	13.22	15	70
Mathematics achievement	95.48	13.63	70	134
**KINDERGARTEN**				
Word reading	112.11	13.11	84	158
Numerical operations	106.63	11.83	75	146

N = 100 for all tasks.

**Table 3:** **Correlations of tasks assessed**.

		**1**	**2**	**3**	**4**	**5**	**6**	**7**	**8**	**9**	**10**	**11**	**12**	**13**	**14**	**15**	**16**
**DOMAIN-GENERAL ABILITIES**																	
1	Y1 EF	–															
2	Y2 EF	0.41^***^	–														
3	IQ	0.41^***^	0.40^***^	–													
4	Preliteracy	0.22^*^	0.29^**^	0.30^**^	–												
**QUANTITATIVE TASKS**																	
5	Y1 GN	0.45^***^	0.54^***^	0.44^***^	0.44^***^	–											
6	Y1 NR	0.15	0.24^*^	0.28^**^	0.69^***^	0.51^***^	–										
7	Y1 NVC	0.25^*^	0.32^**^	0.33^**^	0.19	0.31^**^	0.20^*^	–									
8	Y1 DQD	0.36^***^	0.35^***^	0.32^**^	0.10	0.36^***^	0.09	0.17	–								
9	Y2 GN	0.38^***^	0.23^*^	0.31^**^	0.37^***^	0.37^**^	0.27^**^	0.24^*^	0.35^***^	–							
10	Y2 NR	0.21^*^	0.28^**^	0.30^**^	0.59^***^	0.41^***^	0.55^***^	0.16	0.20^*^	0.53^***^	–						
11	Y2 NVC	0.22^*^	0.35^***^	0.22^*^	0.26^**^	0.44^***^	0.29^**^	0.36^***^	0.38^***^	0.44^***^	0.33^**^	–					
12	Y2 DQD	0.20	0.28^**^	0.39^***^	0.30^**^	0.40^***^	0.24^*^	0.30^**^	0.38^***^	0.62^***^	0.33^**^	0.48^***^	–				
**PRESCHOOL MATHEMATICS ACHIEVEMENT**																	
13	Y1 TEMA	0.38^***^	0.48^***^	0.51^***^	0.54^***^	0.69^***^	0.60^***^	0.28^**^	0.43^***^	0.46^***^	0.57^***^	0.39^***^	0.46^***^	–			
14	Y2 TEMA	0.19	0.33^**^	0.34^**^	0.55^***^	0.49^***^	0.50^***^	0.27^**^	0.26^**^	0.48^***^	0.67^***^	0.45^***^	0.42^***^	0.68^***^	–		
**KINDERGARTEN ACHIEVEMENT**																	
15	WIAT WR	0.28^**^	0.43^***^	0.34^**^	0.62^***^	0.54^***^	0.57^***^	0.29^**^	0.25^*^	0.39^***^	0.58^***^	0.35^***^	0.33^**^	0.64^***^	0.69^***^	–	
16	WIAT NO	0.26^*^	0.35^***^	0.34^**^	0.46^***^	0.50^***^	0.43^***^	0.34^**^	0.43^***^	0.39^***^	0.47^***^	0.49^***^	0.45^***^	0.54^***^	0.48^***^	0.64^***^	–

*EF, Executive Functions; GN, Give-a-Number; NR, Numeral Recognition; NVC, Nonverbal Calculation; DQD, Discrete Quantity Discrimination; TEMA, Test of Early Mathematics Ability; WR, Word Reading; and NO, Numerical Operations*.

* *p < 0.05*,

** *p < 0.01*,

*** *p < 0.001*.

### Quantitative tasks

The final models are shown in Table 4, and the summary results for the nested model comparisons in Table 5. As shown in the first section of Table 4, children's performance on the give-a-number task improved significantly from the beginning to the end of preschool, *t*_(97)_ = 13.03, *p* < 0.001. More developed executive functions in year 1 were associated with higher give-a-number scores in both years, *t*_(97)_ = 4.08, *p* < 0.001, whereas IQ was only important for year 1, *t*_(97)_ = 2.3, *p* = 0.024. Similarly, preliteracy skills predicted give-a-number performance in both years (*p* < 0.05), but there was a trend for these being more important in the first than second year, *t*_(97)_ = 1.77, *p* = 0.081.

**Table 4:** **Change in quantitative task performance during preschool**.

**Effect**	**Estimate**	***se***	***df***	***t***	***p***
**GIVE-A-NUMBER (MAX VALUE = 6)**					
Intercept	5.57	0.12	96	44.81	0.001
Year					
1	−2.19	0.17	97	−13.03	0.001
2	0.00	–	–	–	–
IQ	0.07	0.14	97	0.53	0.599
EF	0.41	0.10	97	4.08	0.001
Preliteracy	0.27	0.13	97	2.08	0.040
IQ ^*^ Year					
1	0.41	0.18	97	2.30	0.024
2	0.00	–	–	–	–
Preliteracy ^*^ Year					
1	0.31	0.18	97	1.77	0.081
2	0.00	–	–	–	–
**DISCRETE QUANTITY DISCRIMINATION (MAX VALUE = 100)**					
Intercept	86.04	1.49	96	57.82	0.001
Year					
1	−19.24	1.76	97	−10.94	0.001
2	0.00	–	–	–	–
IQ	4.39	1.36	97	3.23	0.002
EF	0.67	1.61	97	0.42	0.677
Preliteracy	3.37	1.56	97	2.16	0.033
EF ^*^ Year					
1	3.49	1.81	97	1.93	0.056
2	0.00	–	–	–	–
Preliteracy ^*^ Year					
1	−3.89	1.81	97	−2.15	0.034
2	0.00	–	–	–	–
**NONVERBAL CALCULATION (MAX VALUE = 100)**					
Intercept	44.96	1.80	97	24.94	0.001
Year					
1	−21.66	2.19	99	−9.91	0.001
2	0.00	–	–	–	–
Age	4.22	1.44	99	2.94	0.004
IQ	5.19	1.44	99	3.61	0.001
**NUMERAL RECOGNITION (MAX VALUE = 15)**					
Intercept	9.76	0.32	99	30.94	0.001
Year					
1	−4.99	0.39	98	−12.71	0.001
2	0.00	–	–	–	–
Preliteracy	2.65	0.25	98	10.72	0.001

*Dependent variables are raw scores, and other continuous variables are standardized. EF, Executive Functions. Age, age at beginning of first year of preschool*.

**Table 5:** **Fit statistics for nested models predicting quantitative development during preschool**.

	**−2LL**	**χ^2^**	**Δχ^2^**	**Parameters**	***p***	**BIC**	**ΔBIC**
**GIVE-A-NUMBER**							
1. Full	646.0	0.71	–	12	–	719.7	–
2. Drop Year with Age, IQ and EF interactions	649.7	0.43	−0.28	9	*p* > 0.10	705.0	−14.7
3. Drop Age and Parent Education main effects	654.0	0.76	0.33	7	*p* > 0.10	695.4	−9.6
**DISCRETE QUANTITY DISCRIMINATION**							
1. Full	1630.7	9.27		12	–	1704.4	–
2. Drop Year with IQ, Y1 EF, and parent education interactions	1633.9	8.45	−0.82	9	*p* > 0.10	1684.6	−19.8
3. Drop main effect of Age	1634.2	8.42	−0.03	8	*p* > 0.10	1684.9	0.3
4. Drop main effect of parent education	1637.9	9.61	1.19	7	*p* > 0.10	1679.3	−5.6
**NONVERBAL CALCULATION**							
1. Full	1706.7	7.26	–	12	–	1780.4	–
2. Drop Year with Y1 EF, Age, parent education, IQ, and Preliteracy interactions	1711.8	5.96	−1.30	7	*p* > 0.10	1757.9	−22.5
3. Drop main effect of IQ	1712.4	6.10	0.14	6	*p* > 0.10	1753.8	−4.1
4. Drop main effect of parent education	1714.2	6.54	0.44	5	*p* > 0.10	1746.4	−7.4
5. Drop main effect of Preliteracy	1717.0	7.26	0.72	4	*p* > 0.05	1744.6	−1.8
**NUMERICAL RECOGNITION**							
1. Full	1008.7	3.68		12	–	1064.0	–
2. Drop Year with Y1 EF, Age, IQ, parent education, and Preliteracy interactions	1011.8	3.12	−0.56	7	*p* > 0.10	1057.8	−6.2
3. Drop main effect of Y1 EF	1012.2	3.19	0.07	6	*p* > 0.10	1053.6	−4.2
4. Drop main effect of IQ	1013.4	3.41	0.22	5	*p* > 0.10	1050.2	−3.4
5. Drop main effect of Age	1016.4	3.98	0.57	4	*p* > 0.05	1048.6	−1.6
6. Drop main effect of parent education	1021.9	5.13	1.15	3	*p* > 0.05	1044.9	−3.7

EF, Executive Functions; Age, age at beginning of first year of preschool.

Children also showed improvement on the discrete quantity discrimination task, *t*_(97)_ = 10.94, *p* < 0.001. In contrast to give-a-number, the relation between IQ and discrete quantity discrimination was significant across both years; that is, the main effect was significant with no interaction, *t*_(97)_ = 3.23, *p* = 0.002. Year 1 EF was only important in the first year of preschool, *t*_(97)_ = 1.93, *p* = 0.056. Preliteracy skills were significant overall, *t*_(97)_ = 2.16, *p* = 0.034, and significantly less important for year 1 than year 2, *t*_(97)_ = −2.15, *p* = 0.034. Nonverbal calculation performance also improved across years, *t*_(97)_ = 9.91, *p* < 0.001. Both age at the start of preschool, *t*_(99)_ = 2.94, *p* = 0.004, and IQ, *t*_(99)_ = 3.61, *p* < 0.001, were significantly related to nonverbal calculation performance. Neither of these variables interacted with year, indicating they were important in both years. Finally, children's numeral recognition also improved from the beginning of preschool to the end of preschool, *t*_(99)_ = 12.71, *p* < 0.001, and the only predictor of this change in performance was preliteracy skills, *t*_(98)_ = 10.72, *p* < 0.001.

### Preschool mathematics achievement

The final models are shown in Table 6 and the summary results for the nested model comparisons in Table 7. As shown in Table 6, there was a trend for children's mathematics achievement scores to increase from the beginning to the end of preschool, *t*_(95)_ = 1.89, *p* = 0.062. Give-a-number scores at the beginning of preschool predicted mathematics achievement at the end of preschool, *t*_(95)_ = 2.81, *p* = 0.006, but more strongly in the first than second year, *t*_(95)_ = 2.01, *p* = 0.047. A similar pattern was evident for numeral recognition. Accuracy on the discrete quantity discrimination task at the beginning of preschool did not predict mathematics achievement at the end of preschool, *t*_(95)_ = 1.34, *p* = 0.183, but there was a trend for it to be more strongly related to mathematics achievement at the end of the first than second year of preschool, *t*_(95)_ = 1.70, *p* = 0.095. Preliteracy skill was the only non-quantitative measure that predicted mathematics achievement, *t*_(95)_ = 2.57, *p* = 0.012, with no differences in the prediction of first and second year scores.

**Table 6:** **Predictors of mathematics achievement during preschool**.

**Effect**	**Estimate**	***se***	***df***	***t***	***p***
Intercept	39.96	1.91	90	20.96	0.001
Year					
1	−2.57	1.36	95	−1.89	0.062
2	0.00	–	–	–	–
Age	1.76	1.20	95	1.47	0.145
Parent Education					
No info	−0.90	3.41	95	−0.26	0.792
HS degree	−0.19	2.49	95	−0.08	0.940
College degree	0.00	–	–	–	–
IQ	0.49	1.60	95	0.31	0.761
EF	0.30	1.37	95	0.22	0.829
Preliteracy	3.85	1.50	95	2.57	0.012
Give-a-number	4.81	1.71	95	2.81	0.006
Discrete quantity discrimination	1.90	1.42	95	1.34	0.183
Numeral recognition	4.06	1.78	95	2.28	0.025
IQ ^*^ YEAR					
1	2.65	1.56	95	1.70	0.092
2	0.00	–	–	–	–
Give-a-number ^*^ Year					
1	3.57	1.78	95	2.01	0.047
2	0.00	–	–	–	–
Discrete quantity discrimination ^*^ Year					
1	2.54	1.51	95	1.68	0.095
2	0.00	–	–	–	–
Numeral recognition ^*^ Year					
1	3.19	1.61	95	1.98	0.051
2	0.00	–	–	–	

EF, Executive Functions; Age, age at beginning of first year of preschool.

**Table 7:** **Fit statistics for nested models predicting mathematics achievement during preschool and kindergarten**.

**Model**	**−2LL**	**χ^2^**	**Δχ^2^**	**Parameters**	***p***	**BIC**	**Δ BIC**
**PRESCHOOL MATHEMATICS ACHIEVEMENT**							
**Domain general predictors**							
1. Full	1621.1	37.13		12	–	1694.8	–
2. Drop non-significant interaction (Preliteracy)	1621.1	37.12	−0.01	11	*p* > 0.10	1690.2	−4.6
3. Drop non-significant interaction (EF)	1622.4	36.43	−0.69	10	*p* > 0.10	1686.9	−3.3
4. Drop non-significant interaction (parent education)	1624.6	35.23	−1.20	9	*p* > 0.10	1679.8	−7.1
**Add Quantitative predictors**							
1. Model 4 above and Quantitative predictors	1556.7	18.76		17	–	1648.8	−
2. Drop non-significant interaction (Age)	1557.8	18.29	−0.47	16	*p* > 0.10	1645.3	−3.5
3. Drop non-significant interaction (NVC)	1558.8	17.90	−0.39	15	*p* > 0.10	1641.7	−3.6
4. Drop main effect (NVC)	1561.1	18.83	0.93	14	*p* > 0.10	1639.4	−2.3
**KINDERGARTEN ACHIEVEMENT**							
**Domain general predictors**							
1. Full	1467.6	21.90		14	–	1550.5	–
2. Drop non-significant interaction (IQ)	1467.8	21.80	−0.10	13	*p* > 0.10	1546.1	−4.4
3. Drop non-significant interaction (Y1 EF)	1468.1	21.70	−0.10	12	*p* > 0.10	1541.8	−4.3
4. Drop non-significant interaction (Y2 EF)	1469.0	21.27	−0.43	11	*p* > 0.10	1538.1	−3.7
5. Drop non-significant interaction (parent education)	1471.6	20.15	−1.12	10	*p* > 0.10	1531.5	−6.6
**Add Quantitative predictors**							
1. Model 5 above and Quantitative predictors	1445.0	14.85		18	–	1541.7	−
2. Drop non-significant interaction (NR)	1445.3	14.73	−0.12	17	*p* > 0.10	1537.4	−4.3
3. Drop non-significant interaction (NVC)	1445.6	14.62	−0.11	16	*p* > 0.10	1533.1	−4.3
4. Drop non-significant interaction (GN)	1446.5	14.29	−0.33	15	*p* > 0.10	1529.1	−4.0
5. Drop non-significant main effect (NVC)	1448.9	15.18	0.89	14	*p* > 0.10	1527.2	−1.9
6. Drop non-significant main effect (NR)	1451.6	16.23	1.05	13	*p* > 0.05	1525.3	−1.9

EF, Executive Functions; GN, Give-a-Number; DQD, Discrete Quantity Discrimination; NR, Numeral Recognition; NVC, Nonverbal Calculation. Age, age at beginning of first year of preschool.

### Kindergarten achievement

The tasks used to estimate intelligence can be split into nonverbal IQ (block design) and verbal IQ (information and receptive vocabulary). Preliminary analyses indicated that there was no evidence of differential relations between nonverbal and verbal IQ and mathematics and reading achievement. Thus, we used full scale IQ in our final models. The final models are shown in Table 8 and the summary results for the nested model comparisons in Table 7. As shown in Table 8, children had a lower average score on Numerical Operations than Word Reading, *t*_(96)_ = −5.92, *p* < 0.001. The critical findings were that beginning of preschool preliteracy scores predicted kindergarten Word Reading more strongly than Numerical Operations scores, *t*_(96)_ = 2.94, *p* = 0.007, whereas beginning of preschool discrete quantity discrimination accuracy predicted Numerical Operations more strongly than Word Reading scores, *t*_(96)_ = −2.46, *p* = 0.023. In contrast, beginning of preschool give-a-number performance predicted overall WIAT performance, *t*_(96)_ = 2.71, *p* = 0.008.

**Table 8:** **Predictors of kindergarten achievement**.

**Effect**	**Estimate**	**se**	**df**	***t***	***p***
Intercept	57.71	1.43	90	40.35	0.001
Test					
NO	−6.23	1.05	96	−5.92	0.001
WR	0.00	–	–	–	–
Age	1.34	1.04	96	1.29	0.201
Parent Education					
No Information	−1.57	2.55	96	−0.62	0.539
H.S. Degree	0.31	1.85	96	0.17	0.867
College Degree	0.00	–	–	–	–
IQ	0.94	1.05	96	0.90	0.370
Y1 EF	−0.69	1.02	96	−0.68	0.499
Y2 EF	0.93	1.00	96	0.93	0.355
Preliteracy	3.63	1.05	96	3.46	0.001
Give-a-Number	2.95	1.09	96	2.71	0.008
Discrete Quantity Discrimination	3.47	1.05	96	3.31	0.001
Age ^*^ Test					
WR	−1.88	1.07	96	−1.75	0.083
NO	0.00	–	–	–	–
Preliteracy ^*^ Test					
WR	2.94	1.07	96	2.74	0.007
NO	0.00	–	–	–	–
Discrete Quantity Discrimination ^*^ Test					
WR	−2.46	1.07	96	−2.31	0.023
NO	0.00	–	–	–	–

EF, Executive Functions; Age, age at beginning of first year of preschool; NO, Numerical Operations subtest of WIAT; WR, Word Reading subtest of WIAT; Numerical Operations and Word Reading were both scored as raw score/maximum score from our sample to generate an accuracy score. The maximum score for Numerical Operations was 14, and the maximum score for Word Reading was 93.

## Discussion

### Domain-general predictors of quantitative task performance

Children's competence for each of the four key early quantitative skills improved significantly across the preschool years and, with the exception of numeral recognition, overall performance across years or gains in performance was related to one or both of the domain-general abilities of intelligence or executive functions, in keeping with previous studies (e.g., Clark et al., 2010; Geary, 2011; Fuchs et al., 2016). Intelligence was significantly related to first year knowledge of the cardinal value of number words (i.e., give-a-number), accuracy at discriminating sets of discrete quantities, and competence with nonverbal calculations, as well as second year performance for the two latter tasks. The importance of executive functions, controlling intelligence, emerged for give-a-number in both years and discrete quantity discrimination in the first year. It is not surprising that intelligence and executive functions are related to children's discrete quantity discrimination performance, given the results of Fuhs and McNeil (2013), who found that ANS acuity was significantly correlated with inhibitory control. Overall, it appears that the combination of intelligence and executive functions is significantly related to initial, beginning of preschool quantitative competencies but that only one or the other is important thereafter. Because these two domain-general abilities are correlated, leading to collinearity in the regression results, we cannot say with certainty whether one is more important than the other after the first year of preschool, but the pattern suggests an overall reduction in the importance of domain-general abilities across the 2 years (Geary, 2005; Tricot and Sweller, 2014; Sweller, 2015).

Children's preliteracy skill was also significantly related to several quantitative abilities, and especially their recognition of Arabic numerals. The latter is very similar to our alphabet recognition preliteracy measure and both, in theory, should reflect individual differences in the ease of forming visual-verbal associative relations, in keeping with Koponen et al.'s (2013) proposal. Ease of forming these relations might also contribute to children's performance on the give-a-number task; specifically, mapping number words to representations of associated quantities (Rousselle and Noël, 2007). Associative learning, however, would not explain the relation between preliteracy scores and accuracy on the discrete quantity discrimination task. This is because associative learning should not be necessary for the latter task (Feigenson et al., 2004). Moreover, unlike the numeral recognition and give-a-number tasks, preliteracy only predicted accuracy on the discrete quantity discrimination task in the second year of preschool. Whether this finding reflects basic processes, such as visual attention (Anobile et al., 2012), common to letter learning and nonsymbolic quantity discriminations remains to be determined.

### Predictors of preschool mathematics achievement

In keeping with studies of older children (e.g., Geary, 2011; Fuchs et al., 2016), a combination of domain-general and domain-specific competencies predicted preschoolers' mathematics achievement. In the final model, there were trends for intelligence and discrete quantity discrimination performance to predict beginning but not end of preschool mathematics achievement. There is also evidence that intelligence may influence the estimate of the relation between ANS acuity and mathematics achievement across years; dropping the interaction of either variable with year results in the other interaction becoming significant (Table 6). Since there is collinearity between intelligence and executive functions (e.g., inhibitory control), this would be consistent with Fuhs and McNeil's (2013) finding that there was a weak association between ANS acuity and mathematics achievement in a low-income sample, and that this association was influenced by inhibitory control. These results suggest that acuity of the ANS may contribute to aspects of early mathematics achievement, consistent with some previous studies (e.g., Libertus et al., 2011; Mazzocco et al., 2011). Our results however are not definitive (see also vanMarle et al., 2014; Chu et al., 2015) and in the broader literature, the contribution of the ANS to mathematics achievement remains contentious (e.g., De Smedt et al., 2013).

In line with Schneider et al.'s (2016) recent meta-analysis, we found evidence that symbolic quantitative knowledge is relatively more important that nonsymbolic knowledge in predicting early mathematics achievement. In particular, children's understanding of the cardinal value of number words (give-a-number) and their recognition of numerals predicted mathematics achievement across both years of preschool, but were more strongly related to first year than second year mathematics achievement. This would suggest that recognizing numerals and understanding the quantities represented by number symbols serve as a foundation for early mathematics ability. The across-year decline in the importance of cardinal knowledge was related in part to ceiling effects; that is, most children scored near the top of this scale by the end of preschool. The mathematical competencies assessed by the TEMA-3 were also more complex at the end than the beginning of preschool, that is, the children correctly solved more items at the end of preschool. The test thus begins to measure competencies that move beyond numeral recognition and cardinality, but this does not undermine their importance at the start of preschool.

Finally, control of preliteracy scores, parental education, intelligence, and executive functions in these analyses suggests that the results for cardinal knowledge and numeral recognition represent the contributions of domain-specific knowledge rather than ease of associative learning or other domain-general abilities, or informal parental teaching. The latter could of course contribute to individual differences in children's cardinal knowledge and numeral recognition, as suggested by previous studies (Levine et al., 2010; McNeil et al., 2011; Purpura and Reid, 2016). Our point is that the control of parental education, preliteracy, and domain-general abilities suggests that it is this domain-specific knowledge that is critical to later mathematics achievement, regardless of how children acquired this knowledge.

### Predictors of kindergarten achievement

Children in our sample had higher word reading than mathematics achievement at the end of kindergarten, but their mathematics achievement was still in the average range. Unlike previous studies that have found a link between domain-general abilities such as intelligence and executive functions in predicting later mathematics and reading achievement (e.g., Geary, 2011; Fuchs et al., 2016), we did not find such a relation for our sample. Although preschool intelligence was not predictive of reading and mathematics achievement in kindergarten, controlling many other variables, it still contributed to beginning of preschool mathematics achievement and to more specific aspects of quantitative development. For example, intelligence predicted overall performance in the give-a-number, discrete quantity discrimination, and nonverbal calculation tasks across both years of preschool, but was more strongly related to give-a-number performance at the beginning of preschool. Similarly, there was a trend for executive functions to be more strongly related to discrete quantity discrimination performance at the beginning of preschool. The overall pattern supports the view that domain-general competencies may be more important for initial learning, but then decline in importance as children begin to rely on more domain-specific skills (e.g., Geary, 2005),

Consistent with the results for the individual quantitative tasks, children's early preliteracy skills contributed to their overall achievement, both word reading and mathematics. These findings are consistent with studies of older children and the associative learning hypothesis (Koponen et al., 2007, 2013; Fuchs et al., 2016). Critically, however, early preliteracy skills had an effect on later word reading achievement above and beyond the relation to later mathematics achievement. This important interaction is consistent with a domain-specific contribution of early letter knowledge on word reading skills 2.5 years later (Blatchford et al., 1987), controlling multiple other factors.

A similar pattern emerged for the discrete quantity discrimination task, which predicted overall achievement but was relatively less important for word reading than mathematics achievement. As noted, the effects on overall achievement are not likely to be related to associative learning, but could be related to attentional factors above and beyond those captured by our executive functions scale (e.g., Anobile et al., 2013). In any case, the added contribution to the prediction of mathematics achievement is consistent with a relation between ANS acuity and mathematics achievement (Libertus et al., 2011), but given the weaker relation during the preschool years it is possible that symbolic mathematics skills are influencing the acuity of the ANS rather than vice versa (Halberda et al., 2012).

The finding that children's early knowledge of the cardinal value of number words predicted later achievement was not too surprising, given previous results (vanMarle et al., 2014; Chu et al., 2015), but we were surprised that it was not more strongly related to mathematics than word reading achievement. It is possible that the give-a-number task has a strong domain-general component to it, such as ease of concept formation. On the other hand, performance on the task does involve a clear natural language component, understanding the meaning of number words (Le Corre and Carey, 2007), and this may be the source of the result. The resolution of these alternative explanations for our finding will have to await follow up studies.

### Limitations and future directions

There are several limitations that call for caution in interpreting our results. First, although the longitudinal component allows for reasonably strong inferences, the data are still correlational and any definitive conclusions will require experimental follow up studies. Second, our inclusion of multiple quantitative tasks and 3 years of mathematics achievement data relative to one preliteracy task and 1 year of word reading achievement means that the study was better designed (by intention) to identify predictors of mathematics than reading achievement. We attempted to control the most commonly identified domain-general abilities and parental background but this does not mean that we identified all of them. Moreover, we argued that domain-general associative learning mechanisms may contribute to learning in both mathematics and reading, following studies with older children (e.g., Koponen et al., 2013; Fuchs et al., 2016), but we did not measure these mechanisms directly. Despite these limitations, our overall results are consistent with previous studies of both academic domains, and point to a combination of domain-general and domain-specific contributors to preschool children's emerging competencies with mathematics and reading.

## Author contributions

FC contributed to collecting and analyzing the data and writing the manuscript. DG contributed to designing the research, analyzing the data, and writing the manuscript. KV contributed to designing the research and writing the manuscript.

## Funding

The study was supported by grants from the University of Missouri Research Board and DRL-1250359 from the National Science Foundation.

### Conflict of interest statement

The authors declare that the research was conducted in the absence of any commercial or financial relationships that could be construed as a potential conflict of interest.
